# A dissemination strategy to promote relational coordination in the veterans health administration: a case study

**DOI:** 10.1186/s12913-021-07009-8

**Published:** 2021-09-27

**Authors:** Brigid Connelly, Catherine Battaglia, Heather M. Gilmartin

**Affiliations:** 1 Denver/Seattle Center of Innovation for Veteran-Centered and Value Driven Care, VA Eastern Colorado Healthcare System, 1700 N. Wheeling St, Aurora, CO 80045 USA; 2 Health Systems, Management and Policy, University of Colorado, School of Public Health, Aurora, CO 80045 USA

**Keywords:** Social marketing, Dissemination, Relational coordination, Veterans

## Abstract

**Background:**

Large healthcare institutions like the Veterans Health Administration (VA) continually seek best practices to improve clinical care. Relational coordination is an evidence-based organizational theory of communicating and relating to coordinate work and drive performance outcomes. Implementing relational coordination-guided practices can be difficult due to challenges with spreading information across large systems. Using social marketing theory and evidence-based dissemination strategies, we developed an evidence-based dissemination plan to educate and motivate researchers and operational staff to study and implement relational coordination in the VA.

**Methods:**

In this case study, we used the four Ps (product, price, place, promotion) of social marketing theory to develop a 2-phase dissemination strategy. In phase one, we created and distributed relational coordination information and invited VA staff to join the Relational Coordination Research Collaborative. In phase two, dissemination efforts targeted researchers ready to implement relational coordination within existing programs of research. Process and outcome measures included dissemination, engagement and adoption data and a post-project survey. Quantitative results were calculated using descriptive statistics. Survey text responses were analyzed using deductive content analysis and a structured categorization matrix.

**Results:**

Phase one included social media dissemination, virtual and in-person presentations, as well as phone and email communication between project staff and the target audience. In total, 47 VA staff became members of the Relational Coordination Research Collaborative and 27 routinely participated in online research seminars. In phase 2, 13 researchers expressed interest in studying relational coordination and 5 projects were selected to participate. Multiple relational coordination-related trainings and publications originated from this program.

**Conclusions:**

Dissemination approaches that involved personalized, one-on-one efforts (e.g., phone or email) seemed to be more effective at disseminating relational coordination compared to social media or online presentations. Participants in phase 2 agreed that relational coordination should be adopted in the VA but indicated that cost would be a barrier. Results support the importance of evidence-based dissemination planning that address the unique costs and benefits of programs.

## Background

Researchers and clinicians continually develop new evidence-based practices to improve clinical care and patient safety. Relational coordination is a theory about the relational dynamics of coordinating work within and between teams and organizations. Implementation of relational coordination-guided management practices has positively influenced performance outcomes including safety, quality, efficiency, client satisfaction, and worker well-being [1]. In healthcare, relational coordination is a salient component of delivering effective, reliable, highly coordinated, patient centered care as well as a key aspect of high reliability organizing [2, 3]. Assessment of relational coordination is achieved through the Relational Coordination Survey (RC Survey), a validated measure of coordination and team performance. The RC Survey assesses the quality of communication and relationships among roles and can be used for research or to support organizational change [4]. Education and collaboration is available through the Relational Coordination Research Collaborative, which promotes the advancement of relational coordination through a diverse international community of leaders, researchers, and change agents.

Relational coordination is a promising, evidence-based practice to enhance communication and relationships in healthcare. However, there has been limited adoption. A potential reason for this “research to practice gap” is the challenge of spreading information about relational coordination to those who could benefit from its application. Slow diffusion of information is a known barrier to rapid adoption of evidence-based practices [5]. Diffusion is the passive and natural spread of information to a broad audience, such as through word of mouth [6]. Large healthcare organizations, such as the Veterans Health Administration (VA), use person-to-person diffusion of information with some benefit. An alternative to diffusion is dissemination, defined as planned efforts to spread practices through targeted, direct delivery of information to a particular audience, such as healthcare providers or patients [5]. While no single diffusion or dissemination strategy is effective for every situation, diffusion has been shown to be less effective than dissemination when the aim is to consistently and rapidly spread best practices to improve patient care [5].

Dissemination strategies selected and crafted to reach a specific audience can positively impact the adoption of best practices [7, 8]. Marketing theory offers a valuable framework to select dissemination strategies for large healthcare organizations. Marketing is the management process responsible for identifying, anticipating, and satisfying customer requirements profitably [9]. Social marketing applies marketing principles to societal objectives, such as improving the health and welfare of individuals and society, rather than corporate ones [10]. Social marketing is an active process that induces behavior change through deliberate influence and persuasion by leaders, compared to more passive marketing approaches that aim to influence behavior change by bringing awareness to a topic [9]. In healthcare, social marketing theory offers a strategy to focus on influencing voluntary, socially beneficial behaviors and harnessing those behaviors to enact social change [10]. The social marketing approach provides dissemination guidance, including identification of the four Ps of marketing, product, price, place, and promotion, the target audience and objective setting, and the creation, testing, and circulation of materials [10].

The four Ps of marketing can inform decision making around dissemination of evidence-based practices in healthcare [10]. Product refers to the practice, process or innovation being disseminated. The product should be designed for and by end users who know their local context, be they an individual provider, a clinical team, a community, or an organization. Price refers to the cost in time and money to end users for adopting and implementing the product. Price is a driving factor in implementation of evidence-based practices for human and financial resources are limited in healthcare settings. Place is how a target audience receives and accesses product information. The ideal is for audiences to invest minimal effort to retrieve product information. This is achieved when the product is pushed to end users through established and frequently visited communication forums (e.g., publications, meetings). Promotion considers the different methods of communication. These range from low touch methods such as social media, radio and television, and internet messages which subliminally promote products through low involvement processing [11]. High touch methods include distribution of branded promotional materials such as pens, folders, and toolkit binders or academic detailing, which allows face-to-face interaction and discussion [12]. Identification and optimization of the four Ps can inform healthcare dissemination plans and may increase the uptake of evidence-based strategies [10].

This paper details the dissemination strategy for The Relational Coordination in the Veterans Health Administration (RC in VA) program, a national project designed to educate and motivate VA research and operational staff to study and implement relational coordination [13] within their programs of research or areas of operation. The VA Health Services Research & Development (HSR&D) program funded the RC in VA as a one-year pilot with hopes of understanding the impact of team communication and relationships on organizational performance. Given the size and geographic dispersion of VA healthcare facilities, the RC in VA program prioritized bringing together VA researchers and operational leaders to establish processes to study relational coordination across settings, conditions, and populations. The goals of the RC in VA program were to develop knowledge and understanding of relational coordination that results in implementation projects that positively impact patient and provider health and safety. The work supports current VA priorities including a focus on patient centered care, learning healthcare systems and high reliability organizing [14–16].

In line with social marketing theory described above, dissemination of the RC in VA program involved multi-component strategies. This was important given the unique needs of individual VA research and operational teams, the institutional structure of the VA, and the fact that relational coordination is novel, and a less well understood approach to study team relationships and communication. Once the objectives for the RC in VA program were identified, the question remained as to optimal dissemination products, price, placement, and promotion techniques. To determine best practices for dissemination, we considered many factors, including the available resources within the VA, the ideal target audience, and the variable involvement of VA researchers and operational staff in team focused assessment and improvement.

This paper describes dissemination of the RC in VA program, including the successful dissemination efforts that led to adoption of relational coordination into currently funded VA research and operational projects. We also present the dissemination strategies that failed to garner lasting engagement. The value of understanding dissemination of the RC in VA program is not exclusive to this project. Rather this paper highlights the useful lessons and strategies that may be applied to the broad dissemination of a theoretically driven team communication and relationship theory and assessment tool through a large healthcare organization.

## Methods

The RC in VA program was disseminated and implemented over two phases. The dissemination phase focused on creating and distributing relational coordination informational materials nationally to hundreds of VA researchers and operational staff and inviting participants to join the Relational Coordination Research Collaborative. The implementation phase focused on engaging a small group (e.g., 10–40) of VA researchers and staff to establish processes to study relational coordination in healthcare teams through use of the RC Survey in their currently funded research or operations programs. Methods for the two phases are described separately.

### Planning and dissemination phase

The target audience for the initial phase were the hundreds of VA researchers and operational staff from all 50 States. The objective was to introduce relational coordination concepts and research to a broad audience. Informational materials were created by the PhD-trained VA study researcher (HG) and tested on relational coordination experts and local researchers. The group identified the need for creativity in these materials and suggested the inclusion of infographics and logos on all products. Active dissemination occurred by the study lead (HG) who scheduled time to speak on monthly cyberseminars hosted by VA HSR&D and operational programs (e.g., VA Office of Nursing Services) and weekly distribution of informational materials via email or phone to VA change agents [17]. Passive dissemination occurred through sharing of content bi-weekly via VA and Relational Coordination Research Collaborative twitter feeds and posting RC in the VA information on the Relational Coordination Research Collaborative webpage. Social media impact was tracked using Twitter analytic measures (impressions and engagement). Visits to the RC in VA webpage were not tracked. Interested colleagues were to follow instructions included in social media messaging to contact the study leads and/or join the Relational Coordination Research Collaborative. Active and passive dissemination of RC in VA content and membership in the Relational Coordination Research Collaborative was paid by the RC in VA program funds and delivered through existing VA communication channels. A promotion calendar was established to deliver social media content weekly, virtual presentations monthly, and newsletters quarterly. Dissemination and engagement data from the dissemination phase were captured as invitations to present RC in VA content, number of email and phone contacts by study leads, social media impressions and engagements, and new VA members to the Relational Coordination Research Collaborative. These metrics were feasible to collect and are common measures to assess the impact of social marketing-based efforts [11].

### Implementation phase

This phase involved partnering with a small group (e.g., 10–40) VA researchers and operational staff to establish processes to study RC in VA. Initial efforts required the creation of relational coordination language and RC Survey information for regulatory bodies such as local research review committees and institutional review boards. Subsequent efforts focused on garnering support from executive leadership within VA facilities to approve RC in VA research focused on Veterans and employees. The target audience for the implementation phase included researchers and operational staff who were VA employees (as noted by a va.gov email address), were current members of the Relational Coordination Research Collaborative and could incorporate relational coordination into current projects. Trainings were planned for VA staff to learn about relational coordination assessment and interventions and apply them to current projects. Trainings were provided through attendance at a relational coordination workshop at the VA HSR&D annual meeting and the 9th Annual Relational Coordination Roundtable. In addition, these conferences provided VA researchers and staff opportunities to network and present their relational coordination work.

The objective of phase two was to support teams to study relational coordination in their research or operational projects using the RC Survey. The RC Survey is a proprietary instrument administered through RC Analytics [18]. The survey was available to five VA teams. Administrative and copyright costs were paid by RC in VA program funds. Implementation products shifted to individual support for researchers and operational staff submitting a RC in VA application and RC Survey measurement, analytic and reporting support for selected projects. An RC in VA application was placed on the relational coordination website and shared via email with interested parties. Promotion of the RC in VA application occurred using the methods described in the dissemination phase. Adoption data from the implementation phase included the number of applications requested, submitted, and funded, as well as training attendance.

An end of project survey was sent to the five RC in VA research project leads 1 year after program implementation. The survey included options to select from a list of items or respond with text to understand the impact of the dissemination efforts, how participants heard about the RC in VA and reasons for participating. The text items were included to allow participants to expand or offer new information on the RC in VA program. The survey requested suggestions on how to build a community of relational coordination work in the VA, information on the early findings from their RC in VA studies, as well as additional application of relational coordination in the VA. Eighteen months after the RC in VA pilot program was launched, we gathered information on current and future VA projects that are or will be using relational coordination via VA funding lists and communication with RC in VA program participants.

#### Analysis

Two researchers (BC and HG) collected the dissemination, engagement and adoption data from VA researchers and operational staff during both phases of the project. Quantitative data were calculated using descriptive statistics. The text responses in the survey were analyzed using deductive content analysis in Microsoft Excel 16.3. A structured categorization matrix [19] was developed to code the responses based on the survey questions. Only content that fit the matrix of analysis were summarized and reported. Sociodemographic variables of participants were not collected.

## Results

### Dissemination - phase one

During the initial dissemination phase, 51 instances of email and phone communication were exchanged between the study lead (HG) and VA researchers and operational staff. Fifteen separate relational coordination presentations were conducted. Content was shared via Twitter 47 times, receiving 62,139 impressions and 398 instances of engagement. Four articles were written for VA newsletters. In total, 47 VA staff members signed up for the Relational Coordination Research Collaborative and 27 (55%) participated in monthly educational webinars (Table 1).

**Table 1:** Dissemination phase one methods and outcomes

	Outcome	Comments
RC in VA Dissemination Efforts	N	
Targeted emails and phone calls	51	October 2018–September 2019
VA Cyberseminars	10	March 2018–June 2019
In-person presentations	5	
Twitter social media campaign	47	Impressions = 62,139 Engagements = 398
Articles for VA newsletters	4	VA Office of Nursing Services VA Office of Rural Health VA Quality Enhancement Research Initiative Denver VA Research Department
RC in the VA Engagement		
New VA members to Relational Coordination Research Collaborative	49	
VA participation in Relational Coordination Research Collaborative webinars	27	Held monthly

### Implementation - phase two

The implementation phase resulted in 13 requests for RC in VA applications; six (46%) applications were submitted, and five (38%) submissions were selected. Fifteen VA researchers and staff were trained in relational coordination assessment and interventions through workshop attendance. Information on the RC in VA programs were shared at five conference podium presentations and in two publications [20, 21]. Five projects submitted additional grants to continue their work. As of April 2020, 14 VA studies are currently using relational coordination as a guiding framework and 15 projects have written relational coordination assessment into future research proposals (Table 2).

**Table 2:** Implementation phase two outcomes

	Outcome
RC in VA Applications	N
Requests for RC in the VA application	13
RC in VA applications submitted	6
RC in VA studies approved	5
RC in the VA Products	
RC in VA podium presentations or workshops	5
RC in VA related grant proposals submitted	5
RC in VA related publications	2
VA investigators trained in relational coordination assessment and interventions	15
VA studies using relational coordination in 2020	14
VA studies using relational coordination in new grants	15

Key: RC in VA = Relational Coordination in Veterans Health Administration

Survey findings from the five RC in VA projects (*n* = 9) indicated most participants (*n* = 5; 56%) learned about the RC in VA project through word of mouth. One participant read about it on Twitter while three found the program through a VA presentation. Three participants heard about the project through other means. Reasons participants applied for the RC in VA project were to assess how current teams were working together and how relational coordination might affect future work (*n* = 3; 34%), assess for relationships between relational coordination and implementation and evaluation of projects (n = 3; 34%), and relational coordination’s ability to support complex and large-scale studies and assist in organizational transformation (*n* = 2; 22%).

Participants saw additional applications of relational coordination assessment and interventions in VA operations (*n* = 8; 88%). Specifically, participants felt relational coordination could help understand team dynamics, help implement VA initiatives, and improve workflow (*n* = 5; 56%). Two participants felt, given the large, siloed nature of VA hospitals, relational coordination could increase communication and introduce new initiatives or transformations throughout the VA. All participants indicated they saw additional application of relational coordination assessment and interventions in their own programs of research. This included using relational coordination to evaluate implementation of programs with operational or frontline staff and to assess and improve staff engagement.

Participants were asked if the price of the Relational Coordination Research Collaborative annual partnership ($125/year) and administrative costs to use the RC Survey ($500+ per project) would have influenced their plans to use relational coordination. The respondents were split in that 45% (*n* = 4) indicated they would not have used the RC Survey if there was a cost, while 56% (*n* = 5) indicated they would have used the RC Survey and support from RC Analytics had there been a cost. They added it would have depended on the price and if they could have written the cost into existing grants. The majority (89%; *n* = 8) indicated they would not continue with the Relational Coordination Research Collaborative partnership for the VA does not reimburse staff for memberships, meaning individuals would be required to pay for membership in the Relational Coordination Research Collaborative themselves.

## Discussion

This paper described dissemination of the RC in VA program to researchers and operational staff across the United States. Guided by social marketing theory, we presented the dissemination efforts that led to adoption of relational coordination into five currently funded VA research and operational projects. Using the social marketing elements of product, price, place, promotion, we highlighted lessons and strategies that can be applied to the broad dissemination of a theoretically driven team communication and relationship theory and assessment tool through a large healthcare organization.

The products created for the RC in VA program were designed for and by end users who knew the VA context [22, 23]. These included informational content that could be delivered during phone calls, in-person meetings, over cyberseminars, and via social media. We offered VA researchers and staff membership into a worldwide community of relational coordination researchers, access to ongoing education, use of a validated survey tool and analytic support. These products were designed to fit the diverse needs of VA researchers, giving them flexibility in the level of engagement with relational coordination. The success of this approach is supported by the results of our project.

Literature on healthcare interventions suggests that tailoring evidence-based practices or interventions to an individual’s preference or need is likely to improve professional practice or patient outcomes [24]. This is in contrast to the “one size fits all” approach, which has been found to be inconsistent with goals of maximizing patient outcomes, quality of care, and intervention adherence [25]. In the RC in VA program, we split the work into dissemination and implementation phases, which allowed people to ease into the relational coordination work and stop at their level of interest. Some stopped at learning and information collection or engaged in the Relational Coordination Research Collaborative, while others studied the concept and continued to use it in their research.

A useful lesson from our experience was the price of participation in the RC in VA program was a significant factor that influenced both the dissemination and implementation phases. While perception of price fluctuates depending on the value and need of an intervention, price is a significant consideration in the overall determination of whether to implement an evidence-based practice [26]. High prices for any product has been reported as a major barrier to implementation and adoption [27]. We proactively obtained grant funding to support the Relational Coordination Research Collaborative partnership and use of the RC survey, so price was not a barrier. This was a successful dissemination strategy, as noted by respondents’ hesitancy to continue as a partner and use the RC Survey in future work due to cost. However, one respondent indicated they would consider ongoing Relational Coordination Research Collaborative membership and others reported they would continue to use the RC Survey if it could be covered by grant funds. Suggesting the initial no-cost option was necessary to draw researchers to relational coordination and allow them to experiment with the concept and assessment tools at minimal risk. Of the four Ps, addressing the price of a product is the most useful dissemination and implementation strategy. However, even for a low-price product, one must attract an audience through consideration of the place and promotion of materials.

The strategic placement of RC in VA products using established communication channels for this population was another useful dissemination strategy. RC in VA products were pushed to researchers and operational staff during the workday, in meetings they already attended, and through VA communication newsletters and social media they routinely reference. We housed all content on the Relational Coordination Research Collaborative website, so participants could find all program materials in one place [28, 29]. The push marketing approach was effective for it targeted new customers who hadn’t heard about the RC in VA product [30]. This dissemination approach focused on piquing researchers’ interest in a manner that took very little effort on their part. Push marketing as a dissemination strategy can apply in any setting and should utilize multiple platforms (e.g., social media, newsletters, presentations) to ensure communication saturation is achieved and the target audience is reached.

The promotional efforts and results of RC in the VA suggested that, while low-effort promotion via social media and newsletters was effective in reminding the target audience of the program, the most impactful promotional effort was active outreach through personal contact via email, phone calls, presentations, and discussion with change agents. Though time consuming and more effortful, these promotional methods built off existing relationships and created new ones. They facilitated dialogue and moved many from inquiry to action. Our finding is in alignment with previous studies that found that information spreads more rapidly and more accurately through high-trust individuals and many are only willing to distribute online information coming from trusted peers [31–33]. We encourage both low and high touch promotional efforts in any large-scale dissemination effort, for in our case study the two approaches were complementary.

While our case study contributes to the understanding of successful dissemination strategies across a large healthcare organization, the study design was observational with no control group. Due to this, our conclusions are based on the opinion of researchers who utilized the RC in VA resource. We did not engage researchers or operational staff who did not participate in the RC in VA program. Additionally, some of these researchers had been exposed to relational coordination in their research prior to our dissemination efforts.

## Conclusion

Dissemination focuses on spreading information widely. Our findings, which align with social marketing theory, suggest dissemination within healthcare settings should consider product design and price, in addition to placement and promotion. Dissemination of the RC in VA program was successful for we proactively designed the product and pricing to our target audience and local context. Our products were flexible, adaptable, and available at no-cost. Placement of products on established communication channels and promotion using low-effort and relationship-based methods were effective. Our dissemination efforts developed knowledge and understanding of relational coordination that have resulted in multiple Veteran-focused implementation projects that hold potential to positively impact patient and provider health and safety.

## Data Availability

The datasets used and/or analyzed during the current study are available from the corresponding author on reasonable request.

## Abbreviations

HSR&D: VA Health Services Research & Development
RC in VA: Relational Coordination in the Veterans Health Administration
RC Survey: Relational Coordination Survey
VA: Veterans Health Administration

## References

[1] Havens DS, Vasey J, Gittell JH, LIN WT (2010). Relational coordination among nurses and other providers: impact on the quality of patient care. J Nurs Manag.

[2] Gittell JH, Hajjar L (2019). Strengthening patient-centered care in the VHA: a relational model of change. J Intern Med.

[3] Gittell JH (2020). Transforming relationships for high performance.

[4] Gittell JH (2011). Relational coordination: guidelines for theory, measurement and analysis.

[5] Held RF, Santos S, Marki M, Helmer D (2016). Dissemination and implementation of an educational tool for veterans on complementary and alternative medicine: a case study. BMC Complement Altern Med.

[6] Carpenter D, Nieva V, Albaghal T, Sorra J. Development of a planning tool to guide research dissemination. Advances in patient safety: from research to implementation 2005;4:83–91. 21250029

[7] Grimshaw JM, Eccles MP, Lavis JN, Hill SJ, Squires JE (2012). Knowledge translation of research findings. Implement Sci.

[8] Medves J, Godfrey C, Turner C, Paterson M, Harrison M, MacKenzie L, Durando P (2010). Systematic review of practice guideline dissemination and implementation strategies for healthcare teams and team-based practice. Int J Evid-Based Healthc.

[9] Dann S (2010). Redefining social marketing with contemporary commercial marketing definitions. J Bus Res.

[10] Kotler P, Lee N (2008). Social marketing: influencing behaviors for good.

[11] Ferrier A. The advertising effect: how to change behaviour. OUP Catalogue. 2014.

[12] Soumerai SB, Avorn J (1990). Principles of educational outreach ('academic detailing') to improve clinical decision making. Jama..

[13] Gittell JH, Fairfield KM, Bierbaum B, Head W, Jackson R, Kelly M, Laskin R, Lipson S, Siliski J, Thornhill T, Zuckerman J (2000). Impact of relational coordination on quality of care, postoperative pain and functioning, and length of stay: a nine-hospital study of surgical patients. Med Care.

[14] Gittell JH, Hajjar L (2019). Strengthening patient-centered care in the VHA: a relational model of change. J Gen Intern Med.

[15] Atkins D, Kilbourne AM, Shulkin D (2017). Moving from discovery to system-wide change: the role of research in a learning health care system: experience from three decades of health systems research in the veterans health administration. Annu Rev Public Health.

[16] Kilbourne AM, Elwy AR, Sales AE, Atkins D (2017). Accelerating research impact in a learning health care system: VA’s quality enhancement research initiative in the choice act era. Med Care.

[17] Haider M, Kreps GL (2004). Forty years of diffusion of innovations: utility and value in public health. J Health Commun.

[18] Relational Coordination. Relational Coordination Analytics [Internet]. 2020. Accessed 10 Aug 2020. Available from: https://rcanalytic.com/

[19] Elo S & Kyngäs H. The qualitative content analysis process. J. Adv. Nurs. [Internet] 2008 Mar [accessed 2020 Jan 22];62(1):107–115. Available from: https://onlinelibrary.wiley.com/doi/full/10.1111/j.1365-2648.2007.04569.x 10.1111/j.1365-2648.2007.04569.x 18352969

[20] Rosen MI, Martino S, Sellinger J, Lazar CM, Fenton BT, Mattocks K (2020). Access to pain care from compensation clinics: a relational coordination perspective. Fed Pract.

[21] Gilmartin HM, Battaglia C, Warsavage T, Connelly B, Burke RE. Practices to support relational coordination in care transitions: observations from the Va rural transitions nurse program. Health Care Manag Rev. 2021;27. 10.1097/HMR.0000000000000300. 10.1097/HMR.0000000000000300 33181554

[22] Meekers D, Rahaim S (2005). The importance of socio-economic context for social marketing models for improving reproductive health: evidence from 555 years of program experience. BMC Public Health.

[23] Schlegelmilch BB, Chini TC (2003). Knowledge transfer between marketing functions in multinational companies: a conceptual model. Int Bus Rev.

[24] Baker R, Camosso-Stefinovic J, Gillies C, Shaw EJ, Cheater F, Flottorp S, et al. Tailored interventions to overcome identified barriers to change: effects on professional practice and health care outcomes. Cochrane Database Syst Rev. 2010;3. 10.1002/14651858.CD005470.pub2. 10.1002/14651858.CD005470.pub2 PMC4164371 20238340

[25] Easthall C, Barnett N (2017). Using theory to explore the determinants of medication adherence; moving away from a one-size-fits-all approach. Pharmacy..

[26] Sindelar JL, Ball SA (2010). Cost evaluation of evidence-based treatments. Addict Sci Clin Pract.

[27] Anderson JG (2007). Social, ethical and legal barriers to e-health. Int J Med Inform.

[28] Arnaboldi V, La Gala M, Passarella A, Conti M (2016). Information diffusion in distributed OSN: the impact of trusted relationships. Peer-to-Peer Networking and Applications.

[29] McDavitt B, Bogart LM, Mutchler MG, Wagner GJ, Green HD Jr, Lawrence SJ, et al. Peer reviewed: dissemination as dialogue: building trust and sharing research findings through community engagement. Prev Chronic Dis. 2016;13. 10.5888/pcd13.150473. 10.5888/pcd13.150473 PMC4797478 26986541

[30] Wheeler R. When to push when to pull: marketing strategies [internet]. 2020. Accessed 5 Aug 2020. Available from: https://www.fool.com/the-blueprint/push-vs-pull-marketing/

[31] Herzog A. 7 useful insights you can learn from twitter analytics [internet]. 2020. Accessed 8 Aug 2020. Available from: https://business.twitter.com/en/blog/7-useful-insights-twitter-analytics.html

[32] Sermpezis P, Spyropoulos T (2014). Understanding the effects of social selfishness on the performance of heterogeneous opportunistic networks. Comput Commun.

[33] Zhu K, Li W, Fu X (2014). Rethinking routing information in mobile social networks: location-based or social-based?. Comput Commun.

